# Long-term in vivo pharmacokinetics of dexamethasone-loaded cochlear implant electrode carrier dummies with optimized release profiles

**DOI:** 10.1038/s41598-026-36620-0

**Published:** 2026-02-08

**Authors:** Arne Liebau, Bernd Kammerer, Michel Kather, Sören Schilp, Kenneth Mugridge, Susanne Braun, Eric Lehner, Stefan K. Plontke

**Affiliations:** 1 Department of Otorhinolaryngology, Head and Neck Surgery, Martin Luther University Halle-Wittenberg, University Medicine Halle, Ernst-Grube-Str. 40, 06120 Halle (Saale), Germany; 2 Center for Biological Systems Analysis ZBSA, Albert-Ludwigs-University Freiburg, Freiburg, Germany; 3 Hermann Staudinger Graduate School, University of Freiburg, Freiburg, Germany; 4 MED-EL Headquarters, Innsbruck, Austria; 5 MED-EL Deutschland GmbH, Starnberg, Germany

**Keywords:** Intracochlear drug delivery, Controlled release, Dexamethasone, Inner ear, Animal study, Cochlear implant, Diseases, Preclinical research, Drug delivery

## Abstract

Cochlear implants (CIs) are the primary treatment for severe hearing loss. However, despite advances in electrode materials, implantation remains invasive and can cause trauma, inflammation, loss of residual hearing, and vestibular dysfunction. Foreign body reactions may lead to fibrosis, increasing electrode impedance and compromising device performance. To address insertion-related trauma, there is growing interest in developing electrode carriers that deliver drugs locally, such as dexamethasone, which has demonstrated efficacy in both preclinical and clinical settings. This study investigates a novel coating strategy to optimize the perilymphatic concentration–time profile of dexamethasone and compares it to fully loaded silicone rods, in which the drug is incorporated within the silicone matrix. Silicone rods coated with 1.3 µg, 2.6 µg, or 5.2 µg dexamethasone were implanted into the scala tympani of guinea pigs. Drug concentrations were quantified over 84 days using liquid chromatography–mass spectrometry (LC–MS), and sequential sampling assessed distribution along the scala tympani. Coated rods exhibited an initial burst release, followed by a sustained steady-state phase. The 5.2 µg group peaked at 450 ng/ml, decreasing to 50 ng/ml by day 84. The 2.6 µg group showed a similar profile with proportionally lower levels. Sequential sampling at day 42 after implantation revealed that dexamethasone distributed throughout the length of scala tympani, forming a basal-apical gradient. Compared to fully loaded rods, the coated variants achieved comparable peak concentrations with substantially lower total drug amounts and a prolonged burst phase, which may enhance the suppression of the immediate inflammatory response following implantation. The improved pharmacokinetic efficiency likely also indicates a safer drug exposure profile.

## Introduction

Cochlear implantation is a well-established intervention for the treatment of acquired and congenital profound hearing loss. However, the procedure remains inherently invasive and can cause insertion trauma. Despite ongoing advancements in the mechanical design of electrode carriers that have progressively reduced the severity of insertion-related damage, surgical manipulation and mechanical irritation during implantation still elicit inflammatory responses^1,2^. These immediate mechanical and subsequent inflammatory effects may lead to the loss of residual low-frequency hearing due to apoptosis of the remaining hair cells in the apical region of the cochlea^3–5^. Over time, insertion trauma may also contribute to degeneration of spiral ganglion neurons throughout the cochlea, thereby compromising CI efficacy as these neurons constitute the essential interface for electrical signal transduction^6^.

Beyond acute inflammation, the cochlear implant provokes a foreign body response that leads to progressive fibrosis around the electrode array and within the scala tympani^7^. This fibrotic tissue acts as an electrical insulator, increasing the impedance of the stimulation electrodes^8^. To compensate for the elevated impedance, higher stimulation currents are necessary, which in turn reduce the dynamic range of the hearing prosthesis, as loudness perception depends on current levels^9^. Moreover, the extent to which stimulation current can be increased is limited not only by the electrical properties of the device but also by the emergence of leakage currents at higher stimulation intensities. These leakage currents broaden the electrical field, thereby reducing the spatial precision of frequency-specific stimulation of spiral ganglion neurons.

Furthermore, progressive fibrosis can disrupt the delicate microarchitecture of the cochlea, particularly affecting the mechanical properties of the basilar membrane and altering intracochlear pressure dynamics. These changes impair the natural stimulation of hair cells, contributing to the gradual loss of residual low-frequency hearing, even when the hair cells responsible for the corresponding frequency range in the apical region remain intact^10–12^.

Improving hearing rehabilitation with CI while minimizing insertion trauma and the associated inflammatory response remains a central research objective. One promising approach involves the development of drug-eluting electrode carriers capable of delivering localized, sustained release of anti-inflammatory, immunosuppressive, and homeostasis-stabilizing agents such as glucocorticoids. Among these, dexamethasone has demonstrated therapeutic efficacy in numerous preclinical and early clinical studies^8,13–16^.

In a previous animal study, we demonstrated that dexamethasone-loaded electrode carrier dummies enabled sustained drug release, maintaining stable perilymph concentrations for several weeks post-implantation^17^. Precise control of drug concentration over time is critical to achieve therapeutic levels while minimizing overall drug exposure. Moreover, aligning drug concentrations with the distinct phases of the insertion trauma may further enhance therapeutic outcomes. An ideal release profile would feature an initial burst to counteract acute inflammation, followed by sustained maintenance levels, and a controlled termination phase that adheres to regulatory requirements for implantable drug delivery systems. This study aimed to optimize the release profile of the dummies by modifying their loading techniques, with the primary goal of prolonging the burst phase and gaining precise control over both the cessation of drug release and the total drug delivered to the inner ear. In this study, we demonstrate that a novel strip-coating technique allows precise tuning of both drug loading and release kinetics. This approach enables the optimization of cochlear pharmacokinetics in alignment with the evolving pathophysiological processes triggered by CI insertion trauma.

## Materials and methods

### Study design

**Fig. 1:**
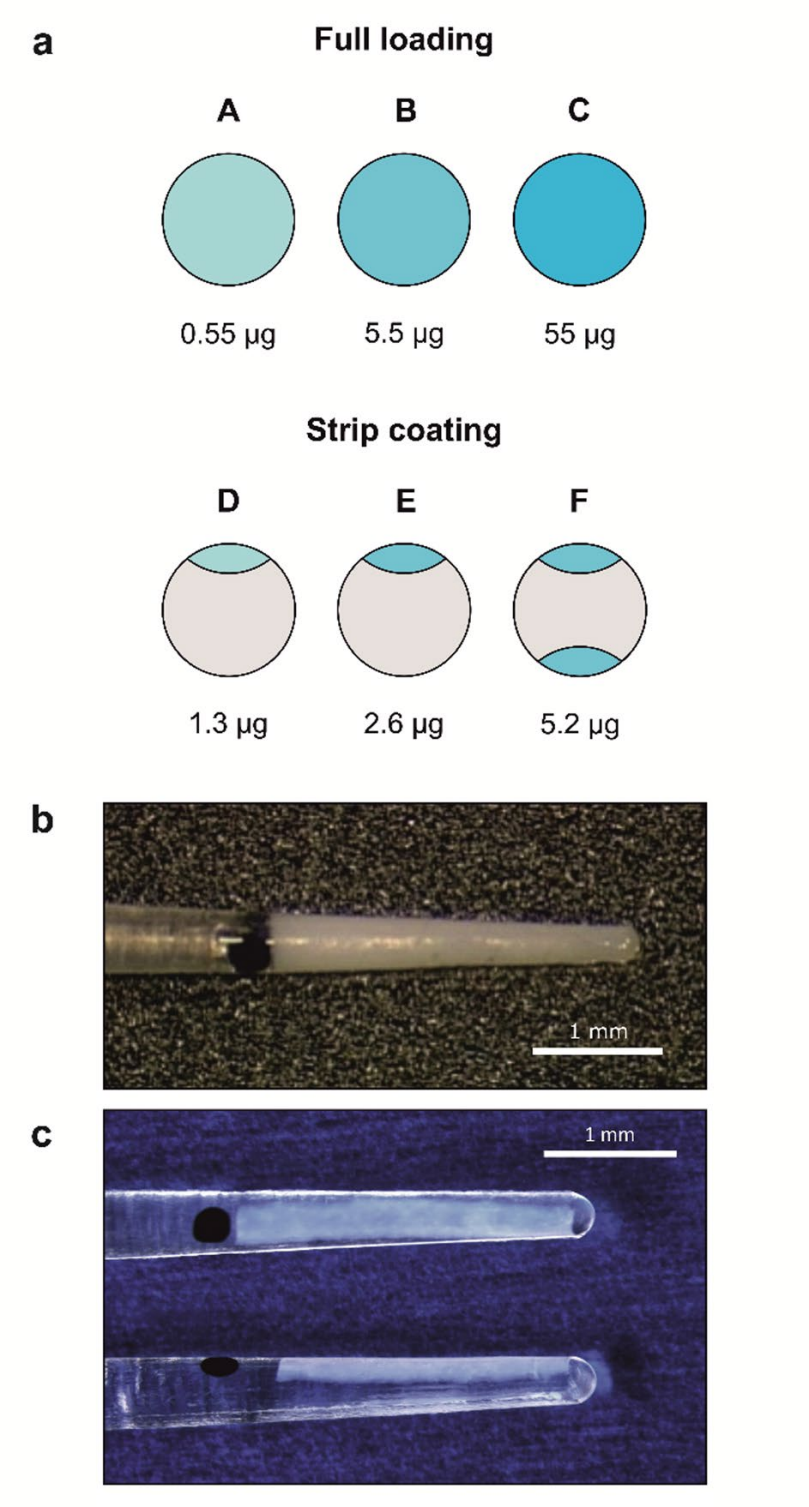
(**a**) Types of loading of electrode carrier dummies with total amount of drug load. A, B, and C represent fully loaded silicone rods from a previous study^17^, where dexamethasone is uniformly distributed throughout the silicone matrix with different concentrations. D, E, and F refer to the silicone rods used in the present study, which are coated with dexamethasone in longitudinal strips, with varying amounts of drug applied. (**b**) Picture of a fully loaded silicone rod of type C. (adapted from Liebau et al. 2020^17^). (**c**) Picture of a strip coated silicone rod of type E.

This study was designed as a descriptive study. Electrode rod-shaped carrier dummies made of silicone were loaded with dexamethasone and implanted into the scala tympani of guinea pigs. Dexamethasone was applied as longitudinal strips on the rods. Three different dexamethasone loading dosages were tested: 1.3 µg, 2.6 µg, and 5.2 µg (Fig. 1a, panels D–F; Fig. 1c). Dexamethasone concentrations in the perilymph were measured at several time points over a period of up to 12 weeks after implantation. Drug concentrations were later compared with those from our previous animal study^17^ using fully loaded silicone rods (Fig. 1a, panels A–C; Fig. 1b).

### Preparation of silicone rods

Silicone rods were manufactured using medical-grade liquid silicone rubber specifically designed for long-term implantation (MED-4244, NuSil, USA). The custom-made rods were adapted to the guinea pig inner ear, with a diameter ranging from 0.3 mm at the tip to 0.4 mm at the base. A black mark on the surface indicated an insertion depth of 3 mm (Fig. 1c). During the coating process a longitudinal hemispherical depression covering the first 3 mm (from tip to base) was created in the silicone rod. This depression was then filled with silicone that had been homogeneously mixed with dexamethasone (C_22_H_29_FO_5_, Sanofi Chimie, Vertolaye, France). Dexamethasone was incorporated in micronized form with crystal sizes below 5 μm. Different dosages of dexamethasone were used in the process which resulted in three different loading levels of the silicone rods: (i) one strip with 1.3 µg (type D), (ii) one strip with 2.6 µg (type E), and (iii) two strips with a total of 5.2 µg (type F) (Fig. 1a). In the third loading variant, type F, a second strip loaded with dexamethasone identical to the one in type E was added, resulting in further doubling of the total amount of drug. The dexamethasone was incorporated into the matrix in crystalline form.

### Implantation of silicone rods

Silicone rods were implanted into the cochleae of 99 female albino guinea pigs (Dunkin-Hartley, Charles River, Wilmington/USA). Animal surgery was performed under general anesthesia, induced by an intramuscular injection (thigh) of a mixture containing medetomidine (0.2 mg/kg), fentanyl (0.025 mg/kg), and midazolam (1 mg/kg). Body temperature was maintained at 37 °C using a heating pad. Additionally, local anesthesia with 1% lidocaine was applied at the incision sites. The surgical procedure for implanting the silicone rods has been previously described in detail^17^. Briefly, implantation was performed in the right ear. The basal turn of the cochlea was accessed through a retroauricular incision and by opening the bulla. A small cochleostomy (0.4 mm) was drilled in the basal turn of the cochlea, 1 mm distal (towards the cochlear apex) from the round window. The silicone rod was then slowly inserted into the scala tympani. The insertion site was dried and sealed with Histoacryl^®^ tissue glue (B. Braun, Melsungen/Germany) around the silicone rod.

### Perilymph sampling

Perilymph samples were collected through apical sampling^18^ at selected time points after implantation (Table 1). Briefly, the cochlear apex was accessed via a ventral approach and by opening the bulla. The cochlear apex was then opened with a small fenestra pick (< 0.1 mm). To measure the average dexamethasone concentration in the scala tympani, the first 5 µl of perilymph seeping from the opening were collected with a glass capillary (type 708707, Blaubrand, Wertheim/Germany). In seven animals implanted with 2.6 µg loaded rods, sequential apical sampling^19^ was conducted on day 42 to assess drug distribution within the scala tympani. For sequential apical sampling, 10 × 1 µl of perilymph were collected with a glass capillary. All samples were diluted to 20 µl with double-distilled water and stored at − 20 °C. After sampling, animals were euthanized with an intracardiac injection of potassium chloride while still under deep anesthesia.

**Table 1:** Number of implanted animals for each type of drug loading of the electrode carrier dummies and time point for Perilymph sampling to measure the average dexamethasone concentration in scala tympani.

Time point after implantation	Drug load		
	1.3 µg	2.6 µg	5.2 µg
Day 1	–	7	7
Day 7	–	7	–
Day 14	–	7	7
Day 28	4	7	7
Day 42	–	7 + 7*	–
Day 56	4	7	7
Day 84	–	7	7

Sequential sampling was conducted at day 42 in seven additional animals implanted with rods loaded with 2.6 µg to measure drug distribution within scala tympani (marked with an asterisk).

### Analysis of the dexamethasone concentration in perilymph

Concentrations of dexamethasone in perilymph samples were determined by liquid chromatography-mass spectrometry (LC-MS). External calibration was performed using analytical standard dexamethasone (CAS 50-02-2). Chromatographic separation of a 5 µl injected sample was achieved with an Agilent Zorbax Eclipse Plus C18 column (2.1 × 50 mm, 1.8 μm) and a ZORBAX Eclipse Plus C18, 2.1 mm, 1.8 μm, UHPLC Guard Column at 20 °C. The mobile phases consisted of water with 0.1% formic acid (Phase A) and acetonitrile with 0.1% formic acid (Phase B). The elution gradient was as follows: 0–8 min, 2–40% B; 8–9 min, 40–98% B; 9–10 min, 98% B; 10–10.1 min, 2% B; 10.1–13 min, 2% B, at a flow rate of 0.5 ml/min. Perilymph samples were analyzed in Multiple Reaction Monitoring (MRM) mode in positive mode using an Agilent 6460 LC-MS/MS system equipped with Agilent Jet Stream ESI Ion Source technology. The lower limit of quantification was 10 ng/ml. Collision energies for all respective dexamethasone fragments were optimized using an Agilent MRM Optimizer. Data were reviewed and processed using Agilent Qualitative (v.B.07.00 SP1) and Quantitative Analysis (v.B.07.01 SP2) software.

## Results

### Perilymph drug concentration over time

**Fig. 2:**
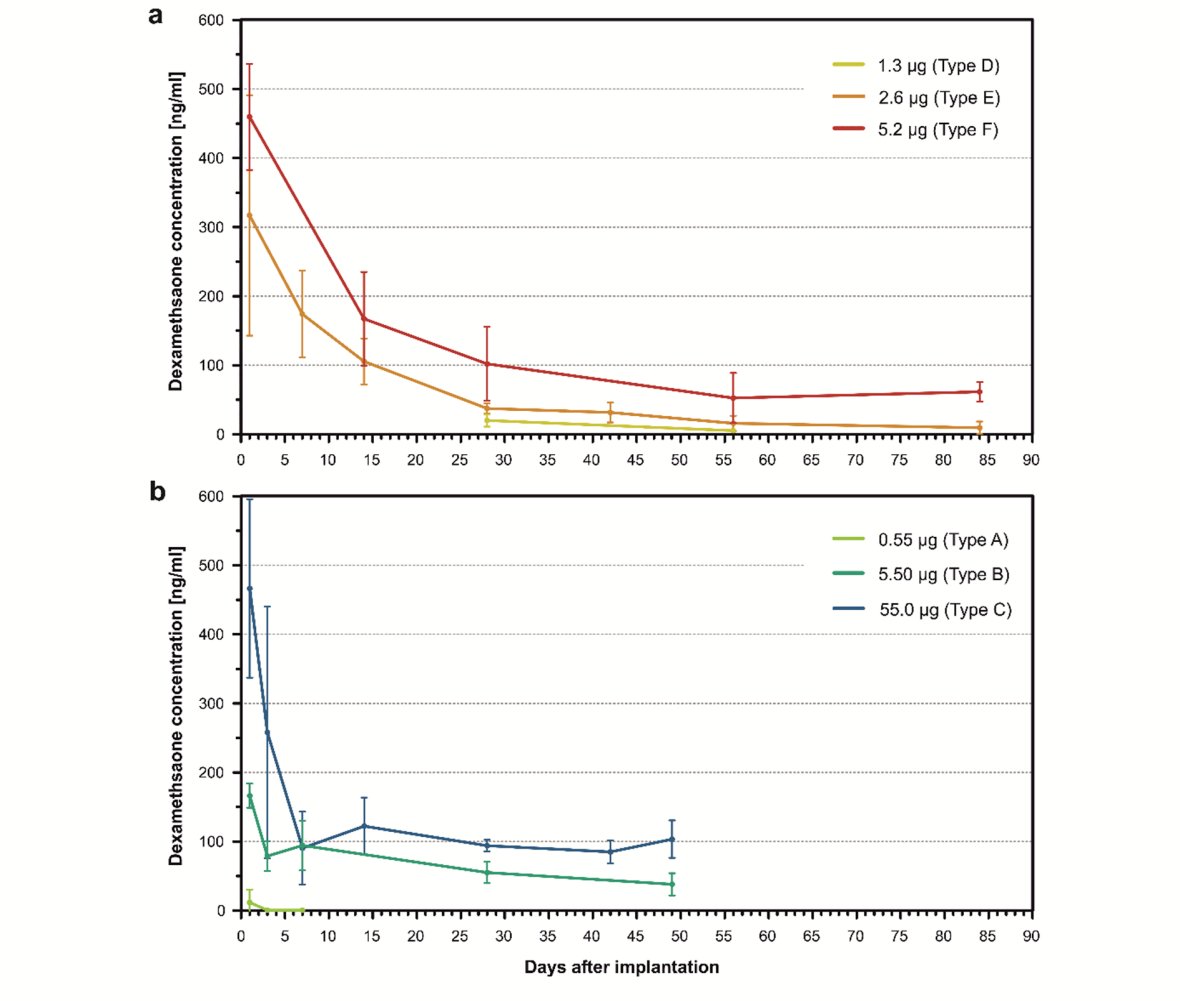
(**a**) Dependence of perilymph dexamethasone concentration in the scala tympani of implanted Guinea pigs with coated dexamethasone-eluting silicone rods (1.3 µg, 2.6 µg, and 5.2 µg) over time after implantation. Each data point represents the mean concentration ± SD (1.3 µg: *n* = 4, 2.6 µg: *n* = 7, 5.2 µg: *n* = 7). A burst release phase lasting 20–40 days, dependent on the drug loading, was followed by a steady-state phase characterized by constant drug concentrations. Second Data point for the 1.3 µg group was below the limit of quantification (LOQ). (**b**) Dependence of perilymph dexamethasone concentration in the scala tympani of implanted animals with fully loaded dexamethasone-eluting silicone rods (0.55 µg, 5.5 µg, and 55.0 µg) over time after implantation (from Liebau et al. 2020^17^) Each data point represents the mean concentration ± SD (0.55 µg: *n* = 3, 5.5 µg: *n* = 3, 55.0 µg: *n* = 3). Data points for the 0.55 µg group were below the limit of quantification (LOQ). The burst release phase in the coated rod types is observed to be more extensive and longer-lasting compared to the fully loaded rod types.

The implantation of rods loaded with 5.2 µg dexamethasone resulted in an initial burst release, with a mean maximum drug concentration of approximately 450 ng/ml one day post-implantation. This concentration gradually decreased over a month, transitioning into a steady-state phase with drug levels initially at approximately 100 ng/ml and later at approximately 50–60 ng/ml (Fig. 2a). No further decline was observed until the end of the 84-day observation time. Rods loaded with 2.6 µg dexamethasone produced an initial burst release, with a mean maximum concentration of approximately 300 ng/ml, with concentrations gradually transitioning to a steady-state phase over nearly a month. At 28 days post-implantation, drug concentrations were approximately 35 ng/ml, decreasing to 10 ng/ml by 84 days. Rods with the lowest loading of 1.3 µg resulted in very low drug concentrations, decreasing from 20 ng/ml to approximately 5 ng/ml (below LOQ) between 28 and 56 days. It became evident that this loading type remained well below the desired concentration range of approximately 50 ng/ml over several weeks. Therefore, no further time points were scheduled for this type. Figure 2b illustrates the perilymph drug concentrations over time for the fully loaded silicone rod type from our previous study^17^, as discussed in the Discussion section.

### Drug distribution in the scala tympani

**Fig. 3:**
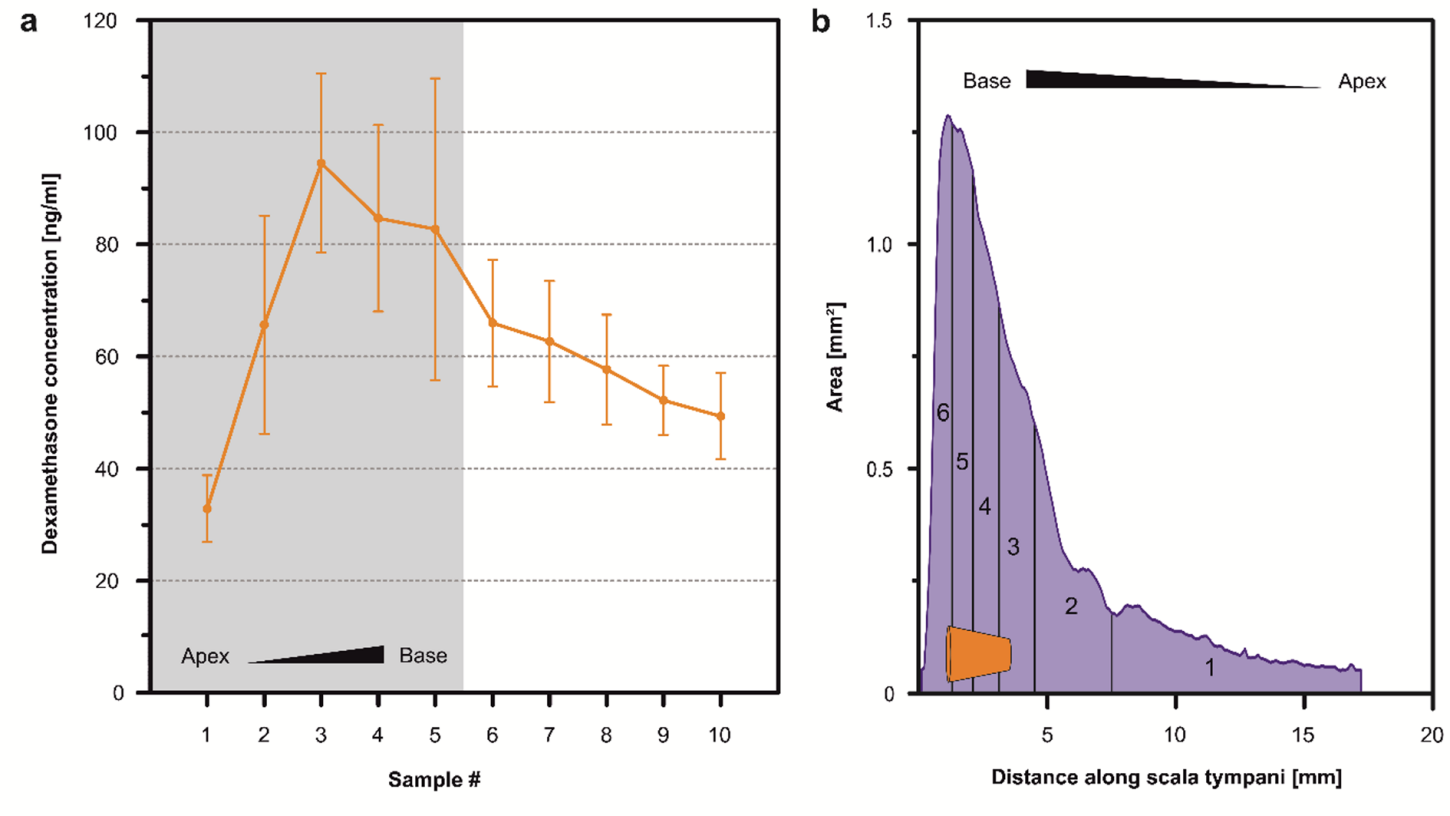
(**a**) Perilymph dexamethasone concentrations after sequential apical sampling (10 × 1 µl) from animals implanted with coated dexamethasone-eluting silicone rods (2.6 µg) on day 42. Each point represents the mean concentration ± SD (*n* = 7). The shaded area represents the perilymph volume of the scala tympani, which is between 5 and 6 µl^20^. The white area indicates the region where perilymph is mixed with cerebrospinal fluid (CSF) from the cochlear aqueduct. The drug is distributed throughout the entire scala tympani, showing a concentration gradient from base to apex. (**b**) Schematic illustration of the scala tympani volume as a function of cross-sectional area in dependence of distance from the basal end of scala tympani with the position of the implanted silicone rod indicated in orange. The rod is introduced via a cochleostomy located 1 mm distal from the round window with an insertion depth of 3 mm. The individual sections correspond to the volumes of the first six 1 µl samples from sequential apical sampling. Data of dimensions of scala tympani taken from FluidSim 5.0 (https://alecsalt.com).

Sequential apical sampling was performed in 10 × 1 µl steps using the 2.6 µg dexamethasone-loaded rods at day 42 post-implantation. Each 1 µl sample can be assigned to spatial regions along the cochlea, with sample #1 corresponding to the apical end and higher-numbered samples representing progressively more basal regions. The most basal region is represented by samples #5–#6 (Fig. 3b). Samples #6 to #10 correspond to cerebrospinal fluid entering the cochlea via the cochlear aqueduct. For the 2.6 µg dexamethasone-loaded rods on day 42, drug concentrations were detected in all sequential apical samples, with a basal-to-apical concentration gradient observed (Fig. 3a). The highest concentration was measured in the basal part of the cochlea (mean: 94.5 ± 9.8 ng/ml, sample #3), while the concentrations of the apical samples (apical half with respect to the length of scala tympani) were approximately three times lower (mean 32.9 ± 4.6 ng/ml, sample #1).

## Discussion

When glucocorticoids are administered to the inner ear, they are rapidly eliminated from the perilymph due to their high lipid solubility and effective permeability across cell membranes. For dexamethasone, the elimination half-life in the scala tympani has been estimated at 46 minutes^21^. Consequently, continuous local drug delivery is necessary to maintain therapeutic concentrations, especially since the insertion trauma caused by cochlear implantation is a prolonged event rather than a single acute incident. Local drug depots offer a distinct advantage by sustaining therapeutic concentrations at the target site while minimizing systemic exposure. In the approach used here, the function of the CI electrode carrier is expanded to include its use as a drug delivery device.

The primary inflammatory phase following CI insertion lasts for several days^22^. Higher concentrations of dexamethasone may be more effective for suppressing the acute inflammatory processes. Therefore, it is reasonable to aim for higher drug concentrations in the cochlea during this initial phase after implantation. Moreover, a stronger initial drug release enhances the concentration gradient, thereby increasing the effective diffusion rate and promoting rapid tissue distribution. An optimal release profile would therefore, consist of an early burst release with minimal delay, followed by a prolonged steady-state phase characterized by a lower, near-constant release rate.

Under continuous local drug delivery conditions, a specific concentration in the perilymph is achieved at a steady-state equilibrium resulting from the balance between drug supply and its elimination via surrounding tissue and subsequent vascular clearance^23^. The pharmacokinetic characteristics of the drug used are crucial for this process. This includes the diffusion rate and solubility, which affect the release of the drug from the matrix into the perilymph, as well as lipophilicity, which determines its elimination rate^24,25^. Additionally, the method of drug incorporation into the depot matrix and the amount of drug loaded are critical factors. To date, three principal strategies have been utilized for drug-releasing electrode carriers: (i) integration of a fluidic channel system within the carrier, which includes one or more outlets combined with a catheter^26^; (ii) direct incorporation of the drug into the carrier’s silicone matrix^14,15,27^; and (iii) coating the surface of the carrier with a matrix layer that incorporates the drug^28,29^.

In our previous study, dexamethasone crystals were homogeneously mixed into the silicone matrix^17^. Three different loading capacities were tested: a 0.1% loading resulting in 0.55 µg of drug content per rod, a 1% loading resulting in 5.5 µg, and a 10% loading resulting in 55 µg of drug per rod (Fig. 1a, panels A–C; Fig. 1b). In all cases, a burst release phase lasting 1 to 7 days was observed depending on the drug loading. This was followed by a steady-state phase characterized by constant drug concentrations (Fig. 2b). The burst release phase was more pronounced for higher drug loadings, and the concentration during the steady-state phase could be controlled by the loading amount. However, the 0.1% loading was not validated throughout the entire observation time, as it became evident that it failed to achieve sufficient long-term drug concentrations.

The measured drug concentrations were compared with results from studies using comparable silicone rods with identical loading, which also included physiological measurements such as hearing threshold shifts, electrode impedances, hair cell loss, and fibrosis in the scala tympani^13,15,27,30,31^. Based on this comparison, it was concluded that maintaining a mean dexamethasone concentration of approximately 50 ng/ml in the perilymph during the steady-state phase is necessary to achieve effective protection against insertion trauma^17^. However, the therapeutically effective dexamethasone concentration in the actual target tissue is likely much lower. Czock et al. 2005 proposed that in human tissues dexamethasone begins to exert pharmacological effects at concentrations ranging from 1 to 30 ng/ml^32^.

**Fig. 4:**
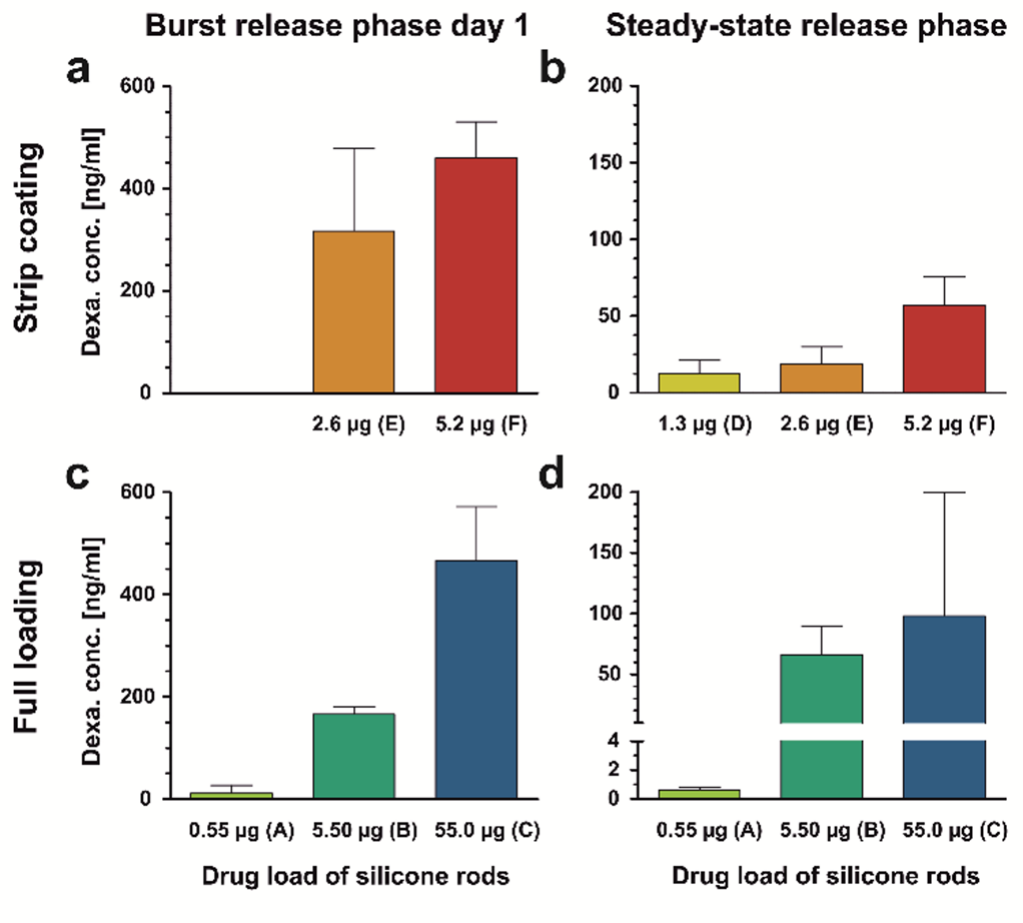
(**a**) Perilymph dexamethasone concentration (mean and SD) in scala tympani during the burst release phase measured on day 1 after implantation for the three tested types of strip-coated rods (see Fig. 2a). *Note* No data was determined for the 1.3 µg type. (**b**) Perilymph dexamethasone concentration (mean and SD) in scala tympani during the steady-state release phase for the three tested types of strip-coated rods. Calculations are based on perilymph drug concentrations at the following time points: 1.3 µg: 28–56 days, 2.6 µg: 42–84 days, 5.2 µg: 56–84 days (see Fig. 2a). (**c**) Perilymph dexamethasone concentration (mean and SD) in scala tympani during the burst release phase measured on day 1 after implantation for the three tested types of fully loaded rods (see Fig. 2b, from Liebau et al. 2020^17^). (**d**) Perilymph dexamethasone concentration (mean and SD) in scala tympani during the steady-state release phase for the three tested types of fully loaded rods. Calculations are based on perilymph drug concentrations at the following time points: 0.55 µg: 3–7 days, 5.5 µg: 3–49 days, 55.0 µg: 7–49 days (see Fig. 2b, from Liebau et al. 2020^17^).

The burst release phase is driven by the drug located on or near the surface of the silicone rod. In contrast, drug concentrations during the steady-state phase are determined by the drug embedded the silicone matrix, which therefore must first diffuse to the rod’s surface. This release mechanism is described by the Higuchi equation, also known as the square root law according to Higuchi^33^, which characterizes the diffusion-driven release of a substance uniformly suspended in an insoluble matrix^34^. The data indicate that perilymph drug concentration shows a hyperbolic-like dependence on the drug loading of fully loaded rods. This relationship exhibited a steeper slope during the burst release phase (Fig. 4c) compared to the steady-state phase (Fig. 4d).

In the current study, a strip-loading drug coating technique was employed (Fig. 1a, panels D–F; Fig. 1c). For both the 2.6 µg and 5.2 µg loading variants, a distinct burst release phase was observed, which gradually transitioned into a steady-state phase after approximately one month (Fig. 2a). During the burst release phase, drug concentrations showed greater variability, which markedly decreased upon entering the steady-state phase (Fig. 4a, b). This increased variability is likely attributable to uneven surface release behavior during the initial phase. As the release shifted to being governed by diffusion from within the matrix, the concentration variance significantly decreased, reflecting more stable and controlled release kinetics.

During the steady-state phase, dexamethasone concentrations ranged from initially 35 ng/ml down to 10 ng/ml by the end of the 84-day observation period for the 2.6 µg loading. For the 5.2 µg loading, concentrations at or above 50 ng/ml were maintained throughout the observation time (Fig. 4b). For both formulations, it is reasonable to assume that drug release continued beyond the final sampling time point. The dexamethasone release profile of the 5.2 µg coated rods mirrored that of the 2.6 µg version, though with consistently higher perilymph concentrations at each time point. The additional drug strip in the 5.2 µg rods resulted in an approximately parallel upward shift of the pharmacokinetic curve.

Compared to fully loaded rods, coated rods exhibited a longer and more gradual burst release phase, with a smoother transition to the steady-state plateau phase (Fig. 2). While the burst release of fully loaded rods was shorter and terminated more abruptly, the coated rods demonstrated a slower decline in drug concentration during this phase. This extended and attenuated release profile may offer improved suppression of the acute inflammatory response triggered by CI insertion trauma. In the coating method, the burst release is similarly governed by drug crystals located on and below the surface. However, unlike fully loaded rods, where the drug is distributed throughout the matrix, the coating confines the drug to a surface-near region. This shortens diffusion pathways and facilitates faster release. As a result, the burst release from coated rods is more pronounced and declines more gradually over time.

Additionally, the particle size of the dexamethasone crystals used in the current study was smaller than the particle size of the previous study with fully loaded rods. Whereas the fully loaded dummies contained micronized dexamethasone crystals of approximately 5 μm, the crystals used for the strip-coated dummies were further reduced to sizes below 5 μm. Smaller crystals dissolve more rapidly than larger particles, according to the Noyes–Whitney equation, leading to a faster drug release^35^. This modification also contributes to the smoother transition between the burst release peak and the steady-state plateau. Furthermore, it results in a faster initial release, producing a more pronounced burst phase.

Implantation of coated rods with 5.2 µg dexamethasone resulted in a burst release comparable to that of the fully loaded 55 µg rods, despite containing nearly ten times less drug. Likewise, coated rods with 2.6 µg dexamethasone produced higher burst release levels than fully loaded rods with 5.5 µg. These findings demonstrate that coated rods can achieve similar or even superior peak perilymph concentrations compared to fully loaded counterparts, while using significantly lower drug quantities. This approach may offer safety advantages by reducing the total drug amount presented to the inner ear. Moreover, concentrating the drug in a defined, surface-near region shortens diffusion pathways and improves predictability. As a result, controlled termination of drug release under clinical conditions becomes more feasible.

To evaluate spatial drug distribution, sequential perilymph sampling was performed during the steady-state phase on day 42 using 2.6 µg coated rods (Fig. 3a). The data reveal that the drug is distributed throughout the entire scala tympani, forming a basal-apical gradient. Given an insertion depth of 3 mm at the cochlear base, the electrode dummies position approximately aligns with sequential perilymph samples #3 to #5 (Fig. 3b), where the highest dexamethasone concentrations were measured, ranging from approximately 80 to 90 ng/ml. A concentration gradient towards the apex is observed, with 30 ng/ml still detectable in the more apical region. From sequential perilymph sample #6 onwards, a significant dilution occurs due to the influx of cerebrospinal fluid (CSF) from the cochlear aqueduct.

Under clinical conditions, dexamethasone is released along the entire length of the CI electrode carrier, so an extended longitudinal diffusion path toward the apex is not required. However, this consideration becomes relevant when preservation of residual low-frequency hearing is desired, as shorter electrode carriers are used, but the drug still needs to reach the apical hair cells responsible for processing lower frequencies^36,37^. On the other hand, the primary site of inflammation and the main source of inflammatory mediators are typically located in the region of the electrode carrier, where mechanical irritation and foreign body reactions are most pronounced. Nonetheless, if apical drug exposure is therapeutically advantageous, the cochlear anatomy may support this. The progressive narrowing of the scala tympani toward the apex could promote a rapid and extensive apical distribution of the drug^38^.

## Conclusion

To our knowledge, this is the first study to evaluate silicone dummies loaded with various potentially therapeutic doses of dexamethasone with respect to intracochlear distribution and time course of intracochlear presence in a significant number of animals. The coating strategy used in this study provides an effective method for controlled drug release into the cochlea, with a desired burst release and an optimized concentration-time profile that surpasses silicone drug carriers where the drug is incorporated throughout the entire matrix.

## Data Availability

The datasets generated during and analysed during the current study are available from the corresponding author on reasonable request.
